# 2026 EASD evidence-based clinical practice guideline for assessing and managing diabetes distress among adults with type 1 diabetes or type 2 diabetes

**DOI:** 10.1007/s00125-026-06840-0

**Published:** 2026-09-15

**Authors:** Jane Speight, Norbert Hermanns, Walther Jensen, Karin Kanc, Thomas Karagiannis, Michelle Law, Andreia Mocan, Femke Rutters, Jackie Sturt, Francesco Zaccardi, Richard I. G. Holt

**Affiliations:** 1 School of Psychology, Deakin University, Geelong, VIC Australia; 2 Institute for Health Transformation, Deakin University, Geelong, VIC Australia; 3 The Australian Centre for Behavioural Research in Diabetes, Diabetes Victoria, Carlton, VIC Australia; 4 Research Institute of the Diabetes Academy Mergentheim (FIDAM), Department of Clinical Psychology and Psychotherapy, University of Bamberg, Bamberg, Germany; 5 Copenhagen, Denmark; 6 jazindiabetes (Diabetes & Me), Diabetes Centre, Ljubljana, Slovenia; 7 Clinical Research and Evidence-Based Medicine Unit, Second Medical Department, Aristotle University of Thessaloniki, Thessaloniki, Greece; 8 Faculty of Health and Life Sciences, University of Exeter, Exeter, UK; 9 Center for Diabetes, Nutrition and Metabolic Diseases, Emergency Clinical County Hospital, Cluj-Napoca, Romania; 10 Amsterdam UMC, location VUmc, Department of Epidemiology and Data Science, Amsterdam Public Health Research Institute, Amsterdam, the Netherlands; 11 Florence Nightingale Faculty of Nursing, Midwifery & Palliative Care, King’s College London, London, UK; 12 Leicester Real World Evidence Unit, Leicester Diabetes Centre, University of Leicester, Leicester, UK; 13 Human Development and Health, Faculty of Medicine, University of Southampton, Southampton, UK; 14 Diabetes Service, University Hospital Southampton NHS Foundation Trust, Southampton, UK

**Keywords:** Diabetes distress, GRADE, Guideline, Mental health, Type 1 diabetes, Type 2 diabetes

## Abstract

Diabetes distress is the emotional burden that arises from living with, and self-managing, diabetes. The aim of this clinical guideline is to optimise the assessment and management of diabetes distress among adults with type 1 diabetes or type 2 diabetes, promoting consistent, person-centred care. The guideline was developed using the EASD Standard Operating Procedure, GRADE methodology and RIGHT reporting standards. A multidisciplinary Guideline Development Panel, including lived experience experts, formulated clinical questions using the PICOT framework. Two clinical questions were addressed: (1) optimal approaches and timing for identifying and exploring diabetes distress; (2) the effectiveness of psychological, psychoeducational, educational, peer support and technology-based interventions for reducing diabetes distress. Good Practice Statements on assessment were informed by de novo realist reviews. GRADE recommendations on management were derived from a commissioned systematic review and meta-analyses of RCTs. For adults with type 1 diabetes or type 2 diabetes, eight Good Practice Statements emphasise routine discussion of emotional well-being, use of validated tools, ongoing monitoring, collaborative care planning, documentation and availability of psychological support. For adults with type 1 diabetes, conditional GRADE recommendations support psychological interventions, continuous glucose monitoring and automated insulin delivery systems for reducing diabetes distress. For adults with type 2 diabetes, conditional GRADE recommendations support psychological, psychoeducational and educational interventions for reducing diabetes distress. The strengths of this guideline include its rigorous methodology and strong stakeholder engagement. Limitations include the scarcity of high-quality trials, particularly in low- and middle-income countries, and exclusion of non-randomised evidence. Implementation will require training and system-level support for integration into routine care, in addition to dissemination through peer-reviewed publications and open access formats.

**Graphical Abstract:**

**Figure:**
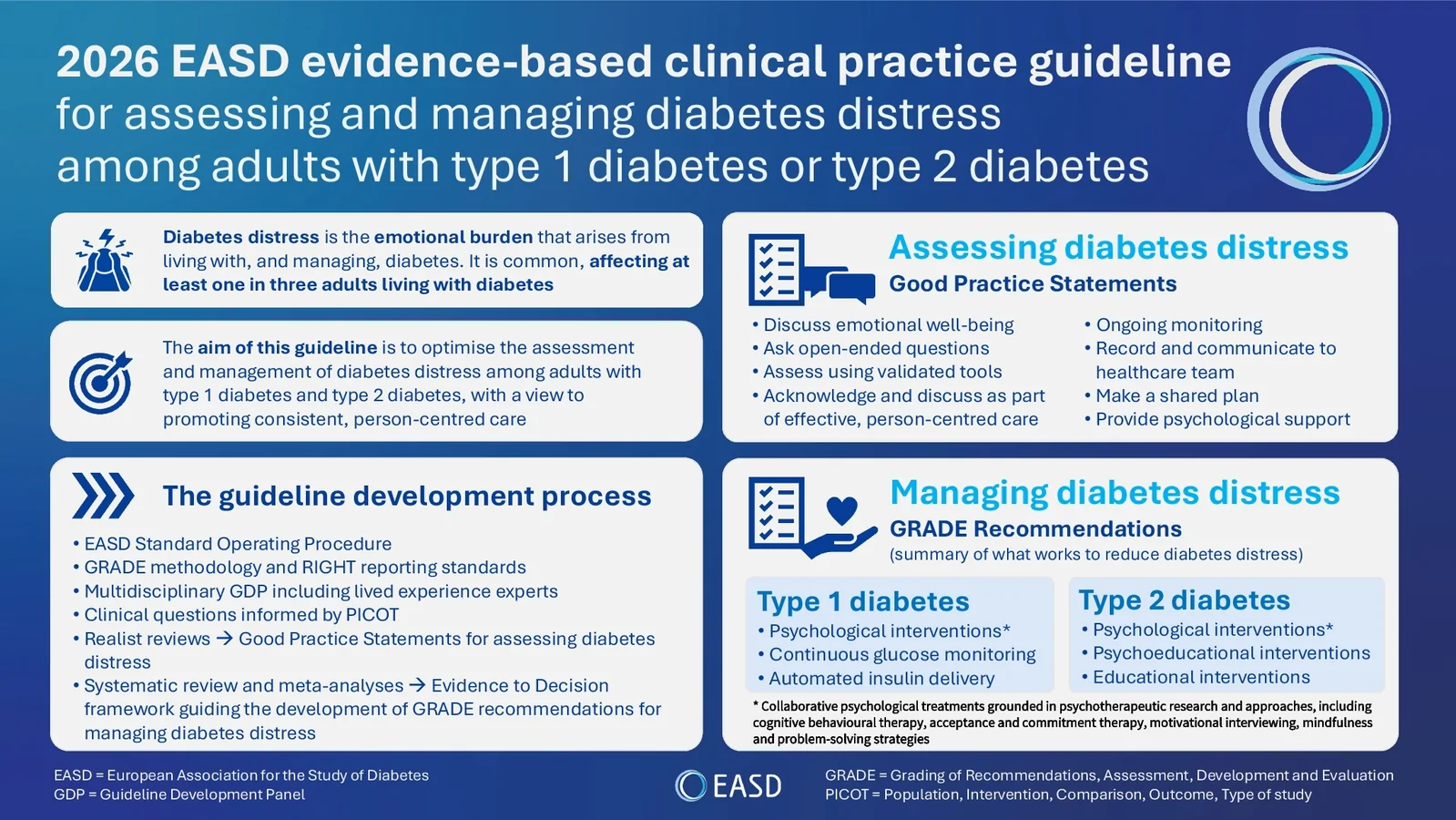

**Supplementary Information:**

The online version contains peer-reviewed but unedited supplementary material available at https://doi.org/10.1007/s00125-026-06840-0.

**Figure:**
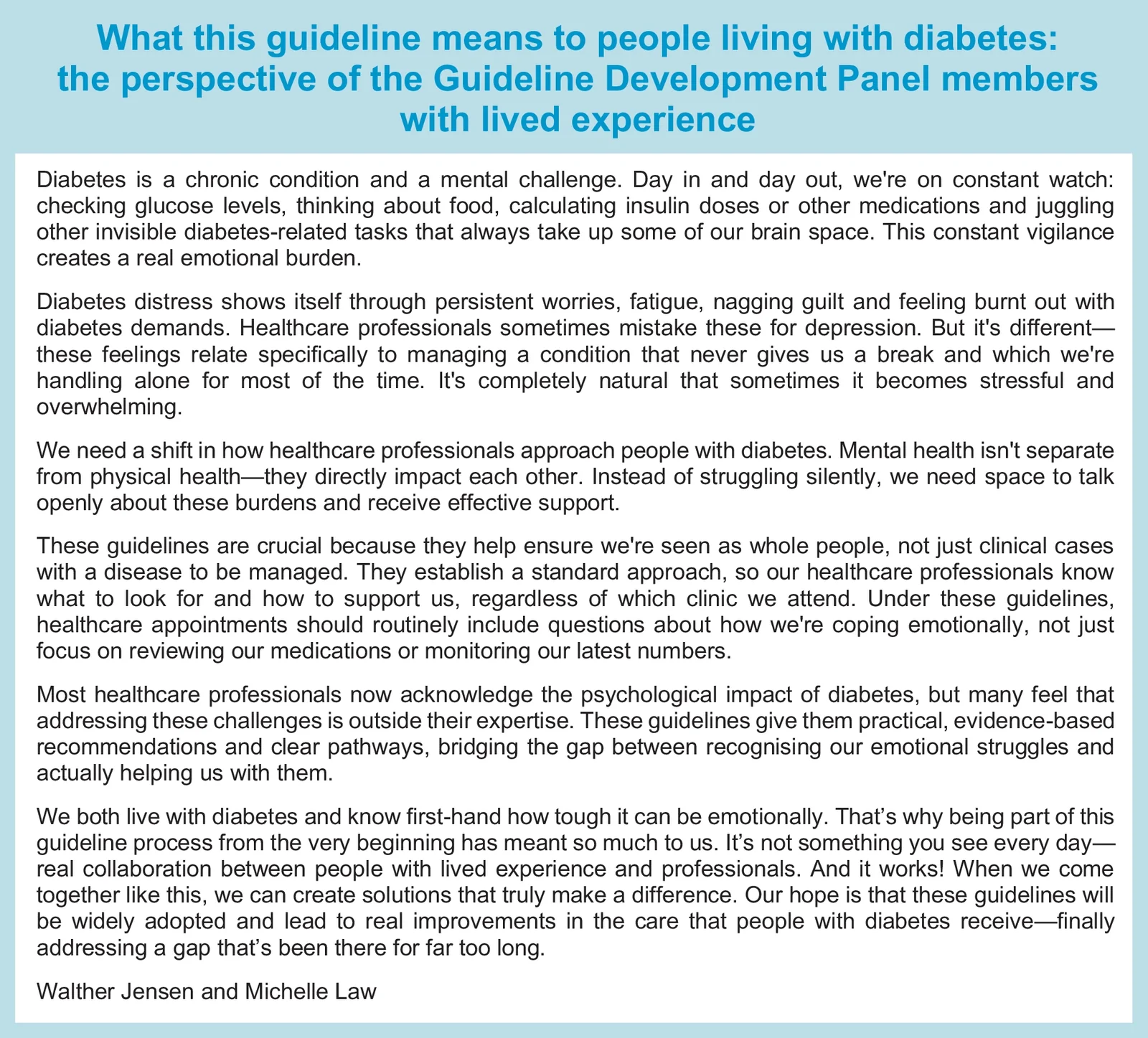

## Introduction

Diabetes distress is the emotional burden that arises from living with, and self-managing, diabetes [1, 2]. As such, it is a part of life when living with diabetes. Adults with type 1 diabetes or type 2 diabetes think about their condition, on average, every 12 min, with frequency varying by treatment type [3]. Diabetes distress can include worries, fears, guilt, frustration and sadness resulting from the everyday management of medications, glucose monitoring, low or high glucose levels, food and physical activity, as well as from other people’s reactions, problem-solving demands and setbacks. In addition to the daily demands of self-care, people living with diabetes need to manage their concerns about or the impacts of serious acute and long-term complications, and navigate social interactions—including curiosity, misinformation and judgement—all of which can further increase the burden.

Diabetes distress is distinct from major depressive disorder, as it represents a specific and natural response to the relentlessness, frustrations and challenges of diabetes. Because these emotional experiences vary widely between individuals, establishing a universal list of defining symptoms—as seen in mental disorders such as depression—is neither appropriate nor clinically meaningful.

### Epidemiology of diabetes distress

Diabetes distress is common among adults with type 1 diabetes. In a longitudinal study, 18% of individuals experienced diabetes distress at baseline, whereas 29% reported elevated distress at least once during the 18 month follow-up [4]. A later study reported a higher prevalence rate of 42% [5]. Population-based data provide additional insights. A Norwegian nationwide registry study of 10,186 adults with type 1 diabetes found that 22% had elevated diabetes distress [6]. Risk factors included being female, younger age, shorter diabetes duration, non-European origin, acute complications (such as diabetic ketoacidosis and severe hypoglycaemia) and CVD. The lower prevalence in the registry study compared with specialist diabetes care settings may reflect differences in the underlying populations. Population-based samples include individuals who are more likely to receive primary care, where emotional challenges related to diabetes may present differently from those in secondary or tertiary care populations.

Diabetes distress is also common among adults with type 2 diabetes. A meta-analysis of 55 studies including 36,998 adults with type 2 diabetes reported an overall prevalence of 36% [7]. Meta-regression identified being female, younger age and comorbid depression as significant moderators associated with higher prevalence. Another study reported marked variation across care settings, with elevated diabetes distress less common in primary care than in secondary care (4% vs 19%) [8]. Additional risk factors beyond those in the meta-analysis include insulin treatment, higher BMI, higher HbA_1c_, the presence of diabetes-related complications and belonging to a minoritised ethnic group [8–11].

Recently, it has been suggested that diabetes distress is more ubiquitous, with up to 80% of adults with diabetes endorsing at least one clinically relevant diabetes problem causing emotional distress [12]. Overall, epidemiological evidence shows that elevated diabetes distress is common in both type 1 diabetes and type 2 diabetes, although prevalence estimates vary substantially across studies, populations and care settings. Diabetes distress may be more prominent or more likely at or soon after diagnosis; when there is a change in treatment regimen; at or soon after diagnosis of complications; or when there is any challenge to coping resources or social support [13]. Awareness of this variability and of the known risk factors may help healthcare professionals identify people with diabetes who are experiencing, or who are at higher risk for, elevated diabetes distress. Such knowledge may facilitate more timely assessment, earlier conversations about emotional well-being, ongoing monitoring and more targeted referral to appropriate psychosocial support when needed.

### Aetiology of diabetes distress

Diabetes distress arises from the ongoing emotional burdens, worries and demands of living with diabetes, but these negative impacts do not affect all individuals in the same way. According to the emotion-regulation framework [2, 14, 15], diabetes distress develops when the emotional demands of diabetes exceed a person’s capacity to identify, interpret and regulate diabetes-related emotions effectively. Thus, the objective burden of the condition, the way individuals experience that burden and the way they manage their emotional responses influence the extent to which distress becomes chronic or overwhelming [14, 16].

Emotion regulation includes conscious and automatic processes that influence emotional experience and expression. When constructive, these mechanisms help people acknowledge feelings and respond deliberately, maintaining a sense of control. Counterproductive patterns such as rumination, avoidance or emotional reactivity amplify negative affect and increase diabetes distress [14]. Empirical evidence supports this pathway, with emotional judgement, reactivity and difficulties in emotional processing predicting diabetes distress [12, 14]. Ineffective emotion regulation, leading to diabetes distress, is associated with suboptimal self-management and worse metabolic outcomes, independent of depressive symptoms [2]. Diabetes distress is more likely when self-management challenges are met with harsh self-evaluation, impulsivity, avoidance or difficulty processing emotions, particularly in the absence of social or professional support.

This framework has two clinical implications: first, interventions that strengthen adaptive emotion-regulation skills may reduce distress and, second, approaches or technologies that reduce the daily diabetes cognitive, emotional or practical burden or uncertainty may lessen the emotional demands that drive counterproductive regulation patterns.

### Impact of diabetes distress on long-term prognosis

Diabetes distress is distinct from depression or other mood disorders, as it is not a diagnosable mental health condition and requires a different therapeutic approach. However, elevated diabetes distress can negatively affect mental health, co-occur with anxiety or depression, and impair self-care and glycaemic indicators [2, 17–19]. If unaddressed, elevated diabetes distress can therefore seriously affect mental and physical health. People with elevated diabetes distress are more likely to develop depression and less likely to recover once depressive symptoms appear [20, 21]. Elevated diabetes distress is a risk factor for reduced quality of life [2, 22], elevated morbidity and premature mortality [23, 24].

Given its impact on mental health and the long-term course of diabetes, timely identification of individuals with elevated diabetes distress is crucial. Early recognition provides an opportunity to prevent more serious mental health problems, reduce the risk of long-term complications or their progression, lower the risk of early death and support better quality of life [2, 7, 13, 25].

### Assessment of diabetes distress

The assessment of diabetes distress can be undertaken through a two-way conversation or by formally quantifying diabetes distress using validated person-reported outcome measures (PROMs). The latter approach is typically used in surveys or RCTs to assess the presence, or severity, of diabetes distress [26]. However, the perspective on assessment presented in this guideline is that of a comprehensive, person-centred approach, characterised by complex interactions that begin before a clinical consultation, by establishing expectations of both the person living with diabetes and their healthcare professional, and continue afterwards, with clinical record keeping and appropriate follow-up.

In clinical consultations, healthcare professionals can assess diabetes distress directly by asking open-ended questions about how individuals are feeling about their diabetes or how they are coping with the everyday challenges or burdens of living with diabetes [27]. This enables individuals living with diabetes to consider and share their recent experiences. Awareness of known risk factors can further help healthcare professionals identify those who may be most vulnerable and direct attention to those who may benefit from closer monitoring or additional support. Importantly, the apparent absence of diabetes distress may not necessarily be the ideal state and needs to be challenged, as it may indicate a lack of awareness, an inability to acknowledge relevant concerns or a reluctance or inability to seek support when needed [27, 28].

In some situations, assessment may warrant the use of validated tools, for example to monitor progress over time or as a conversation starter. Two scales have shaped most diabetes distress research over the past three decades: the Problem Areas in Diabetes (PAID) scale [29] and the Diabetes Distress Scales (DDS) for type 1 diabetes [30] and type 2 diabetes [31]. Several versions of each scale exist, including short-form and contemporary measures. Although there are some differences between these PROMs, which may affect the selection of one over another in a given healthcare setting, all have satisfactory psychometric properties, show strong correlations with each other [32] and are available in several languages. However, not all offer a comprehensive approach to the assessment of diabetes distress. A more detailed description of commonly used assessment tools for diabetes distress is provided in electronic supplementary material (ESM) 1.

### Interventions to address diabetes distress

Importantly, when elevated diabetes distress is identified, healthcare professionals can consider a range of effective interventions [1, 19, 33, 34]. As noted above, elevated diabetes distress can include negative emotions related to treatment regimen, food and eating, low or high glucose levels, worries about the future, social support (interpersonal) and relationships with healthcare professionals. Accordingly, interventions to manage diabetes distress vary in scope, addressing a wide range of needs. Interventions may include psychotherapeutic support, structured education or psychoeducation programmes, organised or ad hoc peer support, the use of technology [33–36] or the simplification or automation of treatment regimens [1].

### Aim of this guideline

The aim of this evidence-based clinical guideline is to support optimisation of comprehensive diabetes distress assessment and management in clinical practice. This guideline is relevant for a broad range of healthcare professionals, organisations and other individuals (see Text box, ‘Who is this guideline for?’). A plain English language summary is available in ESM 2.

**Figure:**
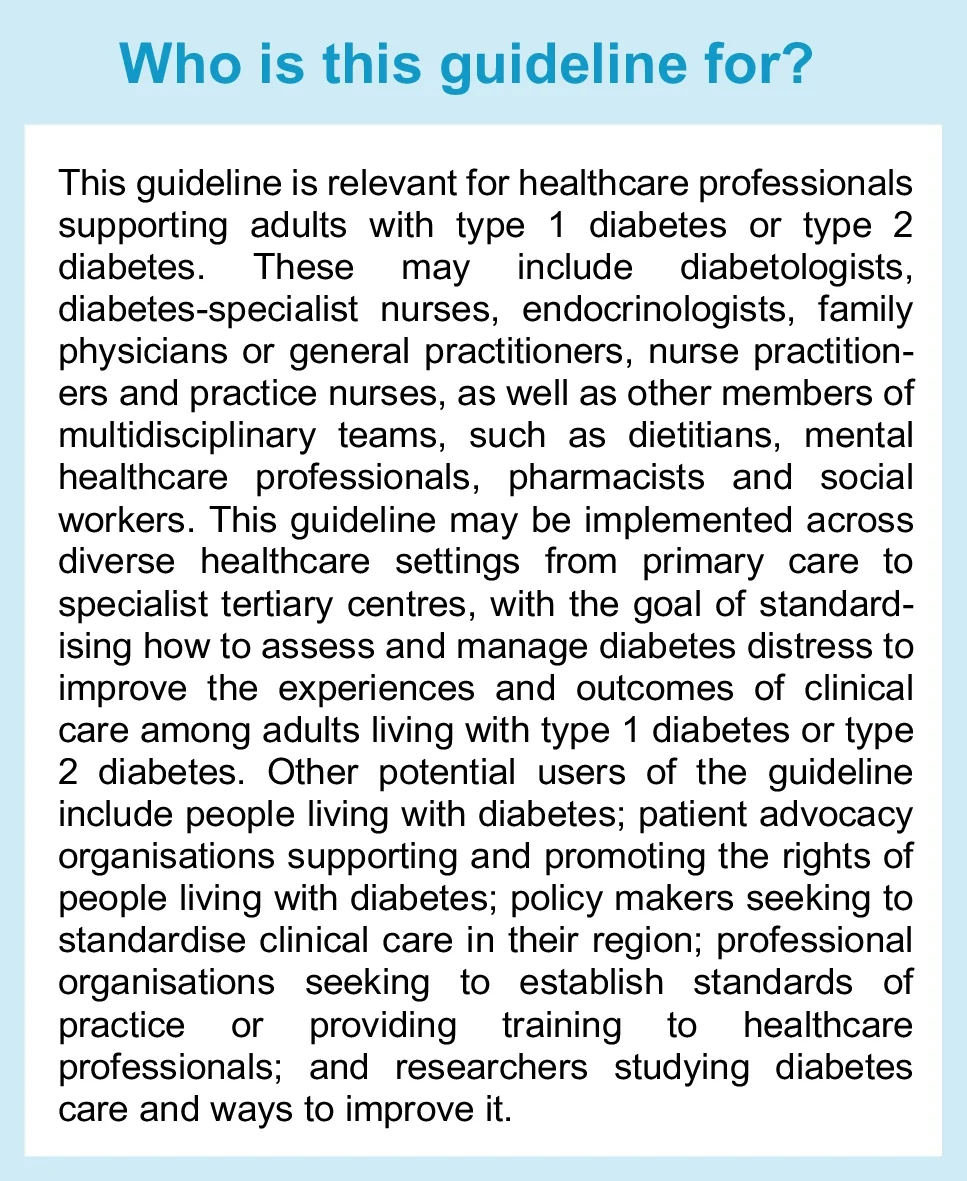

Despite the high prevalence of diabetes distress, and its impacts on self-care, physical health and mental health, there is considerable variation in how healthcare professionals assess and manage it in routine clinical practice. Healthcare professionals report uncertainty about the difference between diabetes distress and depression, about which assessment tools to use and about how to have conversations about diabetes distress, and lack guidance on structured management approaches [12, 27, 37–39]. This leads to missed opportunities to reduce distress and improve diabetes outcomes and quality of life. People living with diabetes also report that the emotional burden of diabetes is overlooked by their healthcare professionals [40–42], resulting in lost opportunities for person-centred care and for identifying solutions to barriers to effective self-management.

These inconsistencies are partly due to the lack of evidence-based guidelines to inform best practice. While ‘Guidelines for encouraging psychological well-being' have existed for over 30 years [43, 44], and recent position statements [45] and practical guides [27] offer support, few are specific to diabetes distress and none include GRADE recommendations based on systematic reviews and meta-analyses. Existing diabetes guidelines include brief recommendations to assess distress but provide little detail about best practice for assessment and management.

This guideline focuses on diabetes distress among adults living with type 1 diabetes or type 2 diabetes. It does not address diabetes distress among children or adolescents, people with gestational diabetes or rarer types of diabetes, or family members of people with diabetes. It also does not cover mental health conditions, such as depression, anxiety or eating disorders, or provide detailed implementation protocols, which are available in a practical guide [27] and training courses. Further details are available from the PsychoSocial Aspects of Diabetes (PSAD) Study Group, a reference group to the EASD on matters related to the psychological, social and behavioural aspects of diabetes (https://www.psadgroup.org/).

This is the first guideline developed under the new EASD guideline development process, according to its Standard Operating Procedure [46] and internationally recognised principles for trustworthy guidelines [47–50].

## Methods

The methods are summarised here. Full details are provided in ESM 3.

### Principles and procedure

The development of this guideline adhered to the standards established by the National Academy of Medicine (NAM) [47], the Guidelines International Network (GIN) [48] and the EASD Standard Operating Procedure for the development of guidelines [46]. Key components included the structured selection of the Guideline Development Panel (GDP), which comprised multidisciplinary experts and individuals with lived experience of diabetes, the transparent management of conflicts of interest, and adherence to GRADE (Grading of Recommendations, Assessment, Development and Evaluation) methodology [49, 50]. The details underpinning these processes have been published [46].

### Guideline Development Panel

The GDP comprised ten members: two co-chairs (JSp, RH), two members with lived experience of diabetes (WJ, ML), four clinical experts (NH, KK, AM, JSt) and two methodologists (TK, FZ). The EASD Guidelines Oversight Committee (GOC) oversaw the appointment and operation of the GDP [46]. The inclusion of two GDP members with lived experience of diabetes was integral to the development of the guideline, ensuring that lived experience was considered in every aspect of the process. All GDP members held equal voting rights and contributed to all stages of the process, including the formulation of clinical questions, review and interpretation of evidence and drafting of recommendations [46, 51].

### Clinical questions

Methodologists conducted PubMed searches to identify existing systematic reviews, which guided the identification and prioritisation of potential clinical questions [52], as well as their relevance to clinical practice, alignment with the priorities of people living with diabetes, and feasibility within the available time frame and resources. Consensus was reached to organise the questions into two broad domains: assessment and management of diabetes distress.

The final set of clinical questions included two assessment questions, one each for adults with type 1 diabetes and adults with type 2 diabetes. Management questions were structured using the PICOT (Participants, Intervention, Comparator, Outcomes, Type of study) framework [52] and developed separately for type 1 diabetes and type 2 diabetes because of differences in aetiology, treatment and lived experience [53, 54].

Interventions were classified as (1) psychological, based on psychotherapeutic approaches such as cognitive behavioural therapy; (2) psychoeducational, which combined psychological components with educational content; and (3) miscellaneous, including education-only interventions, peer support interventions without a psychological component and device-based interventions (e.g. continuous glucose monitoring systems) (see Text box, ‘Definitions of interventions included in the guideline’). Comparators included usual care alone or active interventions from other categories. Validated scales for assessing diabetes distress were reviewed, and a minimal clinically important difference was defined to support interpretation of the intervention effects. Further methodological details are provided in ESM 4 and Schipper et al [55].

**Figure:**
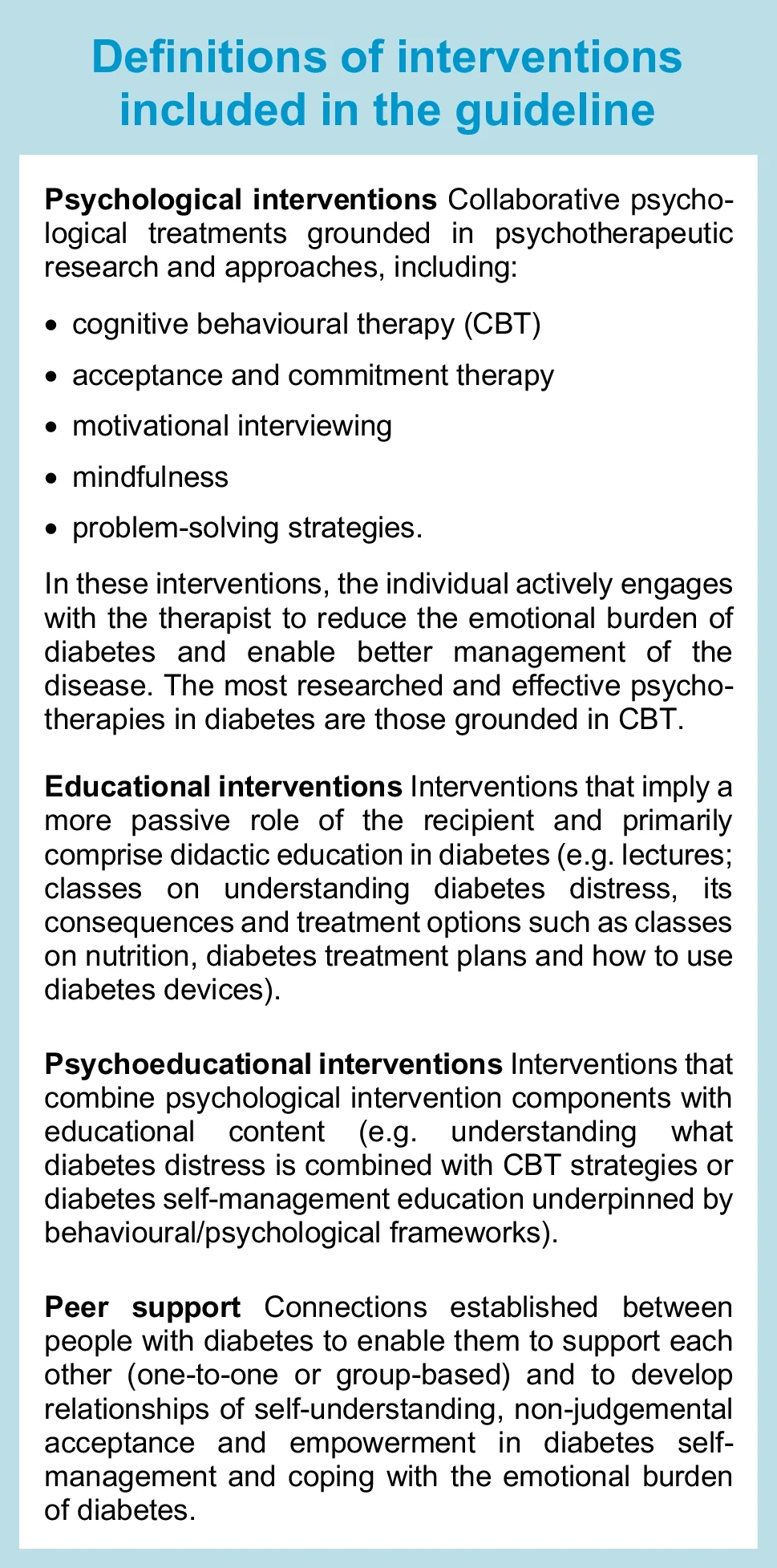

### Assessment of diabetes distress

Assessment questions were addressed using Good Practice Statements [46], as the GDP determined that a traditional evidence synthesis based on RCTs was neither feasible nor appropriate for this topic. The aim was to produce clear, actionable recommendations, reflecting real-world clinical practice, professional consensus and the lived experience of people with diabetes.

As the evidentiary foundation for the assessment guidance, the GDP drew on findings from two de novo realist reviews (one each for type 1 diabetes and type 2 diabetes), conducted as part of the D-Stress project [56, 57]. The reviews used a theory-driven synthesis approach to understand how, for whom and in which contexts approaches to assessing diabetes distress are effective [56, 57].

Based on this information, two GDP members (JSt, JSp) drafted the initial clinical questions, Good Practice Statements and rationales, which were reviewed, discussed and refined by all members of the GDP. The final Good Practice Statements were agreed by expert consensus (see ESM 5).

### Management of diabetes distress

The recommendations on the management of diabetes distress were based on a de novo evidence synthesis of RCTs commissioned by the EASD and were formulated using the GRADE Evidence to Decision (EtD) framework. An independent Evidence Synthesis Team, in collaboration with the GDP, applied standardised systematic review methods, including comprehensive literature searches, screening, data extraction, risk of bias assessment and meta-analyses [55] (see ESM 4).

All included RCTs assessed diabetes distress using validated versions of the DDS or the PAID scales (see ESM 1). To allow comparison across studies, effects were expressed as standardised mean differences (SMDs), with SMDs ≥0.3 considered clinically meaningful.

Certainty of evidence for each meta-analysis was evaluated using the GRADE approach across five domains: risk of bias, inconsistency, indirectness, imprecision and other domains (i.e. publication bias). The two GDP methodologists conducted independent GRADE certainty assessments using GRADEpro GDT [58], resolved differences by consensus and presented the final evidence profiles to the GDP. These profiles informed the construction of the GRADE EtD frameworks, which formed the basis for the recommendations.

The EtD frameworks incorporated not only evidence profiles, but also considerations of benefits and harms, the values and preferences of people living with diabetes, resource use and cost-effectiveness, equity, acceptability and feasibility [59]. Supporting information came from focused literature searches, expert clinical judgement, additional evidence when relevant and the contributions of GDP members with lived experience. The draft EtD frameworks and proposed recommendations were discussed and refined in person using GRADEpro and finalised through consensus. Recommendations were phrased using standardised GRADE terminology to indicate strength and direction.

### Review of the guideline

The draft guideline underwent an internal GDP review and formal EASD GOC review, prior to presentation at the EASD Annual Meeting in Vienna (18 September 2025), followed by an open 6 week public consultation and external peer review. The GDP made revisions following each stage, before final approval by the GOC and EASD Board.

## Clinical practice recommendations

The clinical practice recommendations are categorised as Good Practice Statements for the assessment of diabetes distress (Table 1) or GRADE recommendations for the management of diabetes distress (Tables 2 and 3). Each recommendation is in response to a clinical question and the rationale for the recommendation is summarised, based on the appropriate evidence.

**Table 1:** Good Practice Statements for the assessment of diabetes distress among adults with type 1 diabetes or type 2 diabetes

No.	Recommendation
A.1	Healthcare professionals should discuss the emotional side of diabetes at every consultation as an integral component of person-centred diabetes care.
A.2	Healthcare professionals should ask about diabetes distress using open-ended questions, framed around the emotional burden or challenges of living with diabetes.
A.3	Healthcare professionals should assess diabetes distress using a valid and reliable tool.
A.4	Healthcare professionals should assess and monitor diabetes distress at regular intervals, at least annually, and during significant life events and major treatment changes.
A.5	Healthcare professionals should acknowledge and explore the assessment with the person living with diabetes, irrespective of the findings, as part of effective, person-centred care.
A.6	Healthcare professionals should record the findings of the diabetes distress assessment and discussion in the clinical notes, and communicate them to relevant members of the healthcare team.
A.7	When elevated diabetes distress is identified, healthcare professionals should make a joint plan with the person living with diabetes about next steps.
A.8	General and diabetes-specialist healthcare professionals should have the competency to offer psychological support to those experiencing diabetes distress and know how to enlist specialist support when it is needed.

**Table 2:** GRADE recommendations for the management of diabetes distress among adults with type 1 diabetes

No.	Recommendation	GRADE certainty of evidence
M1.1	We suggest using psychological interventions in addition to usual care, rather than usual care alone, to reduce diabetes distress among adults with type 1 diabetes.	Low ⨁⨁◯◯
M1.2	We do not suggest using psychoeducational interventions in addition to usual care, rather than usual care alone, to reduce diabetes distress among adults with type 1 diabetes.	Very low ⨁◯◯◯
M1.3	We do not suggest using psychological interventions instead of educational interventions or vice versa to reduce diabetes distress among adults with type 1 diabetes.	Very low ⨁◯◯◯
M1.4	We do not suggest psychoeducational interventions instead of educational interventions or vice versa to reduce diabetes distress among adults with type 1 diabetes.	Low ⨁⨁◯◯
M1.5	We suggest using continuous glucose monitoring (CGM), rather than capillary glucose monitoring (finger prick), to reduce diabetes distress among adults with type 1 diabetes.	Moderate ⨁⨁⨁◯
M1.6	We suggest using automated insulin delivery (AID) systems, rather than no use of AID, to reduce diabetes distress among adults with type 1 diabetes.	Moderate ⨁⨁⨁◯

**Table 3:** GRADE recommendations for the management of diabetes distress among adults with type 2 diabetes

No.	Recommendation	GRADE certainty of evidence
M2.1	We suggest using psychological interventions in addition to usual care, rather than usual care alone, to reduce diabetes distress among adults with type 2 diabetes.	Very low ⨁◯◯◯
M2.2	We suggest using psychoeducational interventions in addition to usual care, rather than usual care alone, to reduce diabetes distress among adults with type 2 diabetes.	Very low ⨁◯◯◯
M2.3	We suggest using educational interventions in addition to usual care, rather than usual care alone, to reduce diabetes distress among adults with type 2 diabetes.	Low ⨁⨁◯◯
M2.4	We do not suggest using peer support interventions in addition to usual care, rather than usual care alone, to reduce diabetes distress among adults with type 2 diabetes.	Low ⨁⨁◯◯
M2.5	We suggest using either psychological interventions or educational interventions to reduce diabetes distress among adults with type 2 diabetes.	Very low ⨁◯◯◯
M2.6	We suggest using either psychoeducational interventions or educational interventions to reduce diabetes distress among adults with type 2 diabetes.	Very low ⨁◯◯◯
M2.7	We do not suggest using educational interventions instead of peer support interventions, or vice versa, to reduce diabetes distress among adults with type 2 diabetes.	Low ⨁⨁◯◯
M2.8	We do not suggest using continuous glucose monitoring (CGM), rather than capillary glucose monitoring (finger prick), to reduce diabetes distress among adults with type 2 diabetes.	Very low ⨁◯◯◯

### Assessment of diabetes distress

Detailed results of the realist reviews for type 1 diabetes and type 2 diabetes [56, 57] were used to support the development of Good Practice Statements for the assessment of diabetes distress among adults with either type 1 diabetes or type 2 diabetes.

#### Clinical question A.1

Should healthcare professionals discuss the emotional side of diabetes during routine consultations?

##### Good Practice Statement A.1

Healthcare professionals should discuss the emotional side of diabetes at every consultation as an integral component of person-centred diabetes care.

##### Rationale for the recommendation

Routine discussion of the emotional side of diabetes is important as it (1) acknowledges that diabetes is a challenging and long-term condition and that the emotional side of diabetes is important (for both emotional and physical well-being); (2) ensures that the emotional side of diabetes is valued as an essential component of effective clinical care alongside other metabolic and clinical indicators; (3) prompts acknowledgement and consideration of how the person feels at that time and any unmet needs, enabling insights to be gained into how clinical care can be tailored to support their circumstances and context; and (4) enables early, appropriate intervention to reduce distress related to living with and managing diabetes.

#### Clinical question A.2

How should healthcare professionals ask about diabetes distress in routine consultations?

##### Good Practice Statement A.2

Healthcare professionals should ask about diabetes distress using open-ended questions, framed around the emotional burden or challenges of living with diabetes.

##### Rationale for the recommendation

Routinely asking about diabetes distress with curiosity, and further exploring responses, results in an explicit expectation among both the healthcare professional and the person with diabetes that this is an expected part of usual diabetes care and an important aspect of the healthcare professional’s role.

#### Clinical question A.3

How should healthcare professionals assess for the presence of diabetes distress?

##### Good Practice Statement A.3

Healthcare professionals should assess diabetes distress using a valid and reliable tool.

##### Rationale for the recommendation

This practice enables the healthcare professional and the person living with diabetes to identify specific experiences and sources of diabetes distress. It enables the person living with diabetes to direct the consultation towards issues that are important to them and/or that act as barriers to optimal self-care. The use of valid and reliable assessment tools also ensures consistency in clinical assessments, to facilitate appropriate shared decision-making and development of joint plans, and to monitor changes over time. Validated tools have guidance for scoring and interpretation that can be used to guide whether diabetes distress is substantive enough to require further support or intervention.

#### Clinical question A.4

How often should healthcare professionals assess for diabetes distress?

##### Good Practice Statement A.4

Healthcare professionals should assess and monitor diabetes distress at regular intervals, at least annually, and during significant life events and major treatment changes.

##### Rationale for the recommendation

Assessing diabetes distress as part of the annual cycle of care ensures that it is considered alongside other metabolic and clinical indicators. It also ensures that the emotional side of diabetes is considered during significant life events (e.g. pregnancy), at ‘turning points’ of the condition (e.g. onset or progression of complications) and at major treatment changes (e.g. transition to automated insulin delivery systems) so that no one experiencing diabetes distress is missed.

#### Clinical question A.5

When diabetes distress is assessed, how should healthcare professionals respond?

##### Good Practice Statement A.5

Healthcare professionals should acknowledge and explore the assessment with the person living with diabetes, irrespective of the findings, as part of effective, person-centred care.

##### Rationale for the recommendation

This practice ensures that the person’s experiences of and emotional responses to living with diabetes, whether positive, neutral or negative, are validated and that appropriate actions are considered to support them. Discussion following assessment enables the person living with diabetes to acknowledge their emotional health status. This can help them recognise how they feel in the moment and identify if they need to take action themselves, now or in the future, alongside available or necessary support provided by the healthcare team.

#### Clinical question A.6

How should healthcare professionals communicate the findings of the assessment?

##### Good Practice Statement A.6

Healthcare professionals should record the findings of the diabetes distress assessment and discussion in the clinical notes, and communicate them to relevant members of the healthcare team.

##### Rationale for the recommendation

This practice promotes continuity of care across a multidisciplinary team, ensuring that all members of the team are aware of the experiences of the person living with diabetes. This may be particularly important if the next consultation is with a different member of the healthcare team. This practice enables the person living with diabetes to feel that emotional health is a common thread across the healthcare team, and valued alongside other issues, such as screening and glucose metrics.

#### Clinical question A.7

When elevated diabetes distress is identified, how should healthcare professionals respond?

##### Good Practice Statement A.7

When elevated diabetes distress is identified, healthcare professionals should make a joint plan with the person living with diabetes about next steps.

##### Rationale for the recommendation

This practice enables development of a clear, person-centred plan for reducing diabetes distress. It facilitates a greater understanding of the person’s specific sources of diabetes distress, enabling targeted shared decision-making. Shared follow-up planning may include direct support, referral to appropriate services, and arrangements for timely follow-up care. Joint planning requires healthcare professionals to consider their personal training needs.

#### Clinical question A.8

When diabetes distress is identified, should healthcare professionals deliver psychological support or refer to a mental health professional?

##### Good Practice Statement A.8

General and diabetes-specialist healthcare professionals should have the competency to offer psychological support to those experiencing diabetes distress and know how to enlist specialist support when it is needed.

##### Rationale for the recommendation

This practice is effective and welcomed by people living with diabetes. Having competency extends the scope of the healthcare professional and results in greater role satisfaction.

### Management of diabetes distress

Detailed results of the meta-analyses supporting the clinical practice recommendations for the management of diabetes distress can be found in ESM 4 and Schipper et al [55]. The corresponding GRADE evidence profiles [55] and GRADE EtD frameworks for each management clinical question are provided in ESM 6 and ESM 7, respectively.

### Type 1 diabetes

#### Clinical question M1.1

Should psychological interventions be used, rather than usual care, to reduce diabetes distress among adults living with type 1 diabetes?

##### GRADE Recommendation M1.1

We suggest using psychological interventions in addition to usual care, rather than usual care alone, to reduce diabetes distress among adults with type 1 diabetes.

(Conditional recommendation based on low certainty of evidence)

##### GRADE certainty of evidence

Low certainty evidence ⨁⨁◯◯

This recommendation is conditional, based on low certainty evidence from a meta-analysis of 16 RCTs (17 comparisons) [60–75] including 1343 adults with type 1 diabetes (643 randomised to psychological interventions). The pooled SMD was −0.21 (95% CI −0.34, −0.08), favouring psychological interventions over usual care for reducing diabetes distress.

##### Rationale for the recommendation

We suggest considering psychological interventions alongside usual care, given their small but beneficial effect in reducing diabetes distress and the lack of evidence for potential harm. While the certainty of evidence is low, these interventions appear acceptable to adults with type 1 diabetes, may promote health equity and are consistent with stakeholder values that prioritise emotional well-being. Furthermore, considerations around feasibility and resource use support the potential integration of these interventions into clinical practice.

#### Clinical question M1.2

Should psychoeducational interventions be used, rather than usual care, to reduce diabetes distress among adults living with type 1 diabetes?

##### GRADE Recommendation M1.2

We do not suggest using psychoeducational interventions in addition to usual care, rather than usual care alone, to reduce diabetes distress among adults with type 1 diabetes.

(Conditional recommendation based on very low certainty of evidence)

##### GRADE certainty of evidence

Very low certainty evidence ⨁◯◯◯

This recommendation is conditional, based on very low certainty evidence from a meta-analysis of two RCTs [76, 77] including 133 adults with type 1 diabetes (62 randomised to psychoeducational interventions). The pooled SMD was −0.17 (95% CI −0.62, 0.28) when comparing psychoeducational interventions with usual care for diabetes distress.

##### Rationale for the recommendation

We make a conditional recommendation against the routine use of psychoeducational interventions, as compared with usual care, for reducing diabetes distress among adults with type 1 diabetes. This recommendation is based on evidence of very low certainty derived from two studies. Although the potential for undesirable effects appears to be minimal, the overall balance of benefits and harms does not support the use of psychoeducational interventions. Implementation of such interventions may require additional training for healthcare professionals, as well as integration into routine diabetes care. Feasibility of delivery across diverse healthcare settings may be enhanced by using general or diabetes-specialist healthcare professionals trained in the delivery of such interventions (i.e. a mental health specialist is not necessarily required for intervention delivery) or by using digital delivery formats (where evidence suggests equivalent effectiveness).

#### Clinical question M1.3

Should psychological interventions be used, rather than educational interventions, to reduce diabetes distress among adults with type 1 diabetes?

##### GRADE Recommendation M1.3

We do not suggest using psychological interventions instead of educational interventions or vice versa to reduce diabetes distress among adults with type 1 diabetes.

(Conditional recommendation based on very low certainty of evidence)

##### GRADE certainty of evidence

Very low certainty evidence ⨁◯◯◯

This conditional recommendation is based on very low certainty evidence from a meta-analysis of two RCTs [78, 79] including 482 adults with type 1 diabetes (242 randomised to psychological interventions) and resulting in a pooled SMD of –0.11 (95% CI −0.44, 0.23) comparing psychological interventions with educational interventions for diabetes distress.

##### Rationale for the recommendation

We issue a conditional recommendation not to favour psychological interventions over educational interventions or vice versa for reducing diabetes distress in adults with type 1 diabetes. This recommendation is based on very low certainty evidence from a meta-analysis of two RCTs that found no significant difference between the two intervention types. This recommendation reflects the limited comparative evidence available rather than evidence that one intervention type is more ineffective or harmful than the other. No important differences are expected in terms of undesirable effects, acceptability, feasibility or cost-effectiveness, although these factors may vary depending on context and implementation. It remains uncertain whether psychological interventions offer a greater advantage than educational interventions in reducing disparities; therefore, no conclusions can be drawn regarding their relative impact on health equity.

#### Clinical question M1.4

Should psychoeducational interventions be used, rather than educational interventions, to reduce diabetes distress among adults with type 1 diabetes?

##### GRADE Recommendation M1.4

We do not suggest using psychoeducational interventions instead of educational interventions or vice versa to reduce diabetes distress among adults with type 1 diabetes.

(Conditional recommendation based on low certainty of evidence)

##### GRADE certainty of evidence

Low certainty evidence ⨁⨁◯◯

This conditional recommendation is based on low certainty evidence from a meta-analysis of three RCTs [80–82] including 327 adults with type 1 diabetes (165 randomised to psychoeducational interventions) and resulting in a pooled SMD of –0.02 (95% CI −0.23, 0.20) comparing psychoeducational interventions with educational interventions.

##### Rationale for the recommendation

We make a conditional recommendation not to favour psychoeducational interventions over educational interventions or vice versa for reducing diabetes distress among adults with type 1 diabetes. We found no difference in effect on diabetes distress between the two intervention types, with an almost perfectly symmetrical 95% CI. This recommendation reflects the limited comparative evidence available rather than evidence that one intervention type is more ineffective or harmful than the other. The certainty of the evidence was low and, although some contextual variability is likely, no important differences are expected in terms of undesirable effects, acceptability, feasibility or cost-effectiveness. A conditional recommendation reflects that, while the balance of evidence does not support a clear preference for either intervention, further research may influence this decision. Future studies should explore the comparative effectiveness and consider context-specific factors that may influence implementation and outcomes.

#### Clinical question M1.5

Should continuous glucose monitoring (CGM) be used, rather than capillary glucose monitoring (finger prick), to reduce diabetes distress among adults with type 1 diabetes?

##### GRADE Recommendation M1.5

We suggest using continuous glucose monitoring (CGM), rather than capillary glucose monitoring (finger prick), to reduce diabetes distress among adults with type 1 diabetes.

(Conditional recommendation based on moderate certainty of evidence)

##### GRADE certainty of evidence

Moderate certainty evidence ⨁⨁⨁◯

This conditional recommendation is based on moderate certainty evidence from a meta-analysis of six RCTs [83–88] including 773 adults with type 1 diabetes (425 randomised to CGM). The pooled SMD was −0.26 (95% CI −0.41, −0.11) comparing CGM with capillary glucose monitoring.

##### Rationale for the recommendation

We issue a conditional recommendation for the use of CGM over capillary glucose monitoring in adults with type 1 diabetes, based on moderate certainty of evidence. Evidence from RCTs indicates that CGM results in a small reduction in diabetes distress compared with capillary glucose monitoring. Although undesirable effects such as alert fatigue and information overload may occur, they are unlikely to outweigh the benefits. The cost-effectiveness of CGM appears favourable in certain populations, although this remains context dependent. This conditional recommendation also reflects the variability in values, resources and implementation capacity that may influence decision-making in specific settings. Further research may increase certainty or clarify the impact in subpopulations and under different implementation models.

#### Clinical question M1.6

Should automated insulin delivery (AID) systems be used, rather than no use of AID, to reduce diabetes distress among adults with type 1 diabetes?

##### GRADE Recommendation M1.6

We suggest using automated insulin delivery (AID) systems, rather than no use of AID, to reduce diabetes distress among adults with type 1 diabetes.

(Conditional recommendation based on moderate certainty of evidence)

##### GRADE certainty of evidence

Moderate certainty evidence ⨁⨁⨁◯

This conditional recommendation is based on moderate certainty evidence from a meta-analysis of five RCTs [89–93] including 615 adults with type 1 diabetes (387 randomised to AID). The pooled SMD was −0.15 (95% CI −0.33, 0.02) comparing AID with no AID.

##### Rationale for the recommendation

The available RCT evidence suggests a small, borderline reduction in diabetes distress with AID systems compared with not using AID. Although the effect did not reach conventional statistical significance, the direction of effect favoured AID across studies. Given this pattern of evidence, we issue a conditional recommendation in favour of AID systems for the purpose of reducing diabetes distress in adults with type 1 diabetes. This recommendation applies specifically to the outcome of diabetes distress and does not consider the other potential clinical benefits of AID systems for glycaemic outcomes or additional endpoints outside the scope of this clinical question. Further studies should be designed to assess the impact of AID systems on psychosocial outcomes, with prespecified measures and adequate power to detect meaningful effects on diabetes distress.

### Type 2 diabetes

#### Clinical question M2.1

Should psychological interventions be used, rather than usual care, to reduce diabetes distress among adults with type 2 diabetes?

##### GRADE Recommendation M2.1

We suggest using psychological interventions in addition to usual care, rather than usual care alone, to reduce diabetes distress among adults with type 2 diabetes.

(Conditional recommendation based on very low certainty of evidence)

##### GRADE certainty of evidence

Very low certainty evidence ⨁◯◯◯

This recommendation is based on a meta-analysis of 21 RCTs [63, 65, 67, 71, 73, 74, 94–108] including 4550 adults with type 2 diabetes (2249 randomised to psychological interventions). The pooled SMD for diabetes distress was −0.31 (95% CI −0.50, −0.13), indicating a moderate beneficial effect of psychological interventions compared with usual care.

##### Rationale for the recommendation

We suggest using psychological interventions in addition to usual care because of their moderate beneficial effect on reducing diabetes distress and minimal associated harms. Although the certainty of evidence is very low, such interventions are acceptable to adults with type 2 diabetes, may support equity and align with stakeholder values prioritising emotional well-being. Considerations of feasibility and resource use also support the potential implementation of psychological interventions in clinical practice.

#### Clinical question M2.2

Should psychoeducational interventions be used, rather than usual care, to reduce diabetes distress among adults with type 2 diabetes?

##### GRADE Recommendation M2.2

We suggest using psychoeducational interventions in addition to usual care, rather than usual care alone, to reduce diabetes distress among adults with type 2 diabetes.

(Conditional recommendation based on very low certainty of evidence)

##### GRADE certainty of evidence

Very low certainty evidence ⨁◯◯◯

This recommendation is based on a meta-analysis of 23 RCTs (26 comparisons) [77, 109–130] including 3747 adults with type 2 diabetes (1936 randomised to psychoeducational interventions). The pooled SMD for diabetes distress was −0.19 (95% CI −0.40, 0.02), indicating a small, borderline effect in favour of psychoeducational interventions over usual care.

##### Rationale for the recommendation

We suggest using psychoeducational interventions in addition to usual care because of their potential to provide small reductions in diabetes distress with no evidence of associated harms. Despite the very low certainty of evidence and a borderline effect estimate, the panel judged that the potential benefits, particularly in enhancing patient understanding and coping strategies, outweighed the minimal risks. These interventions may be acceptable to people with type 2 diabetes and align with expressed values around emotional well-being and informed disease management. While feasibility may vary by setting, their integration into routine care remains a reasonable option where resources and personnel permit.

#### Clinical question M2.3

Should educational interventions be used, rather than usual care, to reduce diabetes distress among adults with type 2 diabetes?

##### GRADE Recommendation M2.3

We suggest using educational interventions in addition to usual care, rather than usual care alone, to reduce diabetes distress among adults with type 2 diabetes.

(Conditional recommendation based on low certainty of evidence)

##### GRADE certainty of evidence

Low certainty evidence ⨁⨁◯◯

This recommendation is based on a meta-analysis of three RCTs [131–133] including 317 adults with type 2 diabetes (138 randomised to educational interventions). The pooled SMD for diabetes distress was −0.34 (95% CI −0.56, −0.11), indicating a moderate beneficial effect of educational interventions compared with usual care.

##### Rationale for the recommendation

We suggest using educational interventions in addition to usual care because of their moderate beneficial effect on reducing diabetes distress and minimal associated harms. Although supported by only a few RCTs, the observed effect was meaningful, and there is no evidence suggesting that these interventions are associated with any important harms. Educational interventions may be acceptable to adults with type 2 diabetes and contribute to improved self-management and emotional well-being. While feasibility may vary depending on context, integration into routine care is possible, particularly where training and delivery infrastructure are available.

#### Clinical question M2.4

Should peer support interventions be used, rather than usual care, to reduce diabetes distress among adults with type 2 diabetes?

##### GRADE Recommendation M2.4

We do not suggest using peer support interventions in addition to usual care, rather than usual care alone, to reduce diabetes distress among adults with type 2 diabetes.

(Conditional recommendation based on low certainty of evidence)

##### GRADE certainty of evidence

Low certainty evidence ⨁⨁◯◯

This recommendation is based on a meta-analysis of four RCTs (five comparisons) [134–137] including 815 adults with type 2 diabetes (410 randomised to peer support). The pooled SMD for diabetes distress was −0.03 (95% CI −0.19, 0.13), indicating no meaningful effect of peer support interventions compared with usual care.

##### Rationale for the recommendation

We do not suggest using peer support interventions over usual care for the specific purpose of reducing diabetes distress because the available evidence from RCTs does not demonstrate a clear or clinically meaningful benefit for this outcome. The certainty of evidence is low and the pooled effect estimate was close to null. This finding should be interpreted in the context of the narrowly defined outcome and comparison addressed by this recommendation. Peer support interventions may still provide important benefits for people with diabetes, such as enhanced self-management, greater engagement with care, and improved social and emotional support, outcomes that were outside the scope of this clinical question. It is also plausible that peer support, when combined with other educational or psychological interventions, may contribute to reductions in diabetes distress. Harms of peer support interventions are considered minimal, and feasibility may vary depending on programme design, leadership and integration into existing care systems.

#### Clinical question M2.5

Should psychological interventions be used, rather than educational interventions, to reduce diabetes distress among adults with type 2 diabetes?

##### GRADE Recommendation M2.5

We suggest using either psychological interventions or educational interventions to reduce diabetes distress among adults with type 2 diabetes.

(Conditional recommendation based on very low certainty of evidence)

##### GRADE certainty of evidence

Very low certainty evidence ⨁◯◯◯

This recommendation is based on a meta-analysis of ten RCTs (12 comparisons) [138–147] including 2304 adults with type 2 diabetes (1306 randomised to psychological interventions). The pooled SMD for diabetes distress was −0.29 (95% CI −0.98, 0.39), reflecting a highly uncertain estimate with very low certainty of evidence.

##### Rationale for the recommendation

We suggest that either psychological interventions or educational interventions may be suitable for reducing diabetes distress in adults with type 2 diabetes, given their demonstrated benefits in separate comparisons with usual care. The available evidence does not allow firm conclusions about the relative superiority of one approach over the other, and the wide 95% CIs indicate considerable uncertainty in the estimated effects. Despite this, both interventions are potentially valuable and their implementation could be guided by local context, patient preferences and resource availability.

#### Clinical question M2.6

Should psychoeducational interventions be used, rather than educational interventions, to reduce diabetes distress among adults with type 2 diabetes?

##### GRADE Recommendation M2.6

We suggest using either psychoeducational interventions or educational interventions to reduce diabetes distress among adults with type 2 diabetes.

(Conditional recommendation based on very low certainty of evidence)

##### GRADE certainty of evidence

Very low certainty evidence ⨁◯◯◯

This recommendation is based on a meta-analysis of 13 RCTs [148–160] including 1757 adults with type 2 diabetes (881 randomised to psychoeducational interventions). The pooled SMD for diabetes distress was −0.15 (95% CI −0.34, 0.03), indicating a small, marginally non-significant effect favouring psychoeducational interventions compared with educational interventions.

##### Rationale for the recommendation

The comparative evidence suggests a small, marginally non-significant reduction in diabetes distress for psychoeducational interventions compared with educational interventions, with very low certainty evidence and substantial uncertainty around the magnitude of any difference between the two types of interventions. Importantly, both psychoeducational and educational interventions represent active approaches to diabetes distress management and, within this guideline’s evidence base, each has demonstrated benefit compared with usual care in adults with type 2 diabetes. In this context, and given the overlap in intervention content and variability in implementation, we issue a conditional recommendation supporting either approach. Selection between psychoeducational and educational interventions may reasonably be guided by patient preference, feasibility, availability of trained personnel and the need for structured emotional coping components alongside education.

#### Clinical question M2.7

Should educational interventions be used, rather than peer support interventions, to reduce diabetes distress among adults with type 2 diabetes?

##### GRADE Recommendation M2.7

We do not suggest using educational interventions instead of peer support interventions, or vice versa, to reduce diabetes distress among adults with type 2 diabetes.

(Conditional recommendation based on low certainty of evidence)

##### GRADE certainty of evidence

Low certainty evidence ⨁⨁◯◯

This recommendation is based on a meta-analysis of three RCTs [161–163] including 476 adults with type 2 diabetes (241 randomised to educational interventions). The pooled SMD for diabetes distress was 0.10 (95% CI −0.08, 0.28), for peer support interventions compared with educational interventions, indicating no clear difference between peer support and educational interventions.

##### Rationale for the recommendation

The available head-to-head evidence does not demonstrate a clear difference between educational and peer support interventions in reducing diabetes distress, and the certainty of evidence is low. Although educational interventions demonstrate benefit compared with usual care in this guideline, and peer support interventions do not show a meaningful effect compared with usual care, the direct comparative RCT evidence for this specific question remains limited and does not support a confident preference for one intervention over the other. In light of this uncertainty, we issue a conditional recommendation without favouring one type of intervention over the other. Selection between educational and peer support interventions may reasonably be guided by contextual factors such as patient preference, feasibility, availability of resources and the intended goals of the intervention.

#### Clinical question M2.8

Should continuous glucose monitoring (CGM) be used, rather than capillary glucose monitoring (finger prick), to reduce diabetes distress among adults with type 2 diabetes?

##### GRADE Recommendation M2.8

We do not suggest using continuous glucose monitoring (CGM), rather than capillary glucose monitoring (finger prick), to reduce diabetes distress among adults with type 2 diabetes.

(Conditional recommendation based on very low certainty of evidence)

##### GRADE certainty of evidence

Very low certainty evidence ⨁◯◯◯

This recommendation is based on a meta-analysis of three RCTs [164–166] including 611 adults with type 2 diabetes (345 randomised to CGM). The pooled SMD for diabetes distress was −0.35 (95% CI −0.98, 0.28), indicating no clear benefit of CGM over capillary glucose monitoring for reducing diabetes distress

##### Rationale for the recommendation

We do not suggest using CGM over capillary glucose monitoring solely for the purpose of reducing diabetes distress because of the absence of a demonstrated beneficial effect and the very low certainty of evidence. While CGM may offer important advantages for glycaemic management and diabetes self-management, its specific impact on emotional well-being remains unclear. Implementation should therefore be guided by individual patient needs, preferences and other clinical considerations.

## Recommendations for research

The application of the GRADE framework in this guideline reflects an a priori decision to base management recommendations on RCT evidence on intervention effectiveness. RCTs remain the most reliable design for evaluating causal effects of interventions, including complex interventions, whereas observational studies generally provide lower certainty evidence and are rarely sufficient to support confident conclusions about effectiveness.

Restricting the evidence base to RCTs meant that we were unable to address our original research questions about assessment of diabetes distress. There is thus a need to test care pathways that comprehensively assess diabetes distress in RCTs. We were also unable to identify eligible trials addressing the following original research questions about the management of diabetes distress:

- Should educational interventions, compared with usual care, be used to reduce diabetes distress among adults with type 1 diabetes?
- Should peer support interventions, compared with usual care, be used to reduce diabetes distress among adults with type 1 diabetes?
- Should psychological interventions, compared with psychoeducational interventions, be used to reduce diabetes distress among adults with type 1 diabetes?
- Should psychological interventions, compared with psychoeducational interventions, be used to reduce diabetes distress among adults with type 2 diabetes?

Even when RCT evidence on the management of diabetes distress was available, it was often limited by small sample sizes, heterogeneity of interventions and the frequent use of diabetes distress as a secondary outcome. As a result, the certainty of evidence was low or very low and recommendations appropriately reflect this level of uncertainty. The process of formulating this guideline identified substantial knowledge gaps that should be addressed by RCTs to strengthen future revisions and optimise clinical support and outcomes for adults living with type 1 diabetes or type 2 diabetes. This section outlines priority research directions, organised by thematic area, highlighting both methodological imperatives and substantive knowledge needs.

For clinical questions for which RCT evidence was insufficient to support meta-analysis and GRADE certainty assessment, the GDP considered issuing ungraded clinical practice recommendations [46]. However, to avoid potential misinterpretation, the panel judged it prudent not to do so [167]; instead, the panel identified research priorities to address key evidence gaps, and issued recommendations for research [167]. In the absence of RCT evidence, the panel was unable to make clinical practice recommendations and instead recommended further RCTs to determine the effectiveness of these types of interventions for diabetes distress.

### Education and peer support for adults with type 1 diabetes

Overall, the evidence on education and peer support for diabetes distress in type 1 diabetes suggests considerable and varied benefits [168], but this relies heavily on observational data. No RCTs of education or peer support interventions in adults with type 1 diabetes reported diabetes distress as a primary or secondary outcome. Consequently, there was insufficient evidence to support a GRADE recommendation, in contrast to the evidence available for type 2 diabetes. RCTs are needed to examine the effects of these interventions on diabetes distress in this population compared with usual care. Future RCTs should prespecify diabetes distress as an outcome, use validated instruments and include adequate sample sizes to detect clinically meaningful differences.

### Comparative effectiveness of interventions

Many interventions are multifaceted, and the specific components responsible for reducing diabetes distress remain unclear. Evidence comparing active intervention approaches is sparse and of very low certainty, with existing studies generally underpowered. Direct head-to-head RCTs are therefore needed among adults with type 1 diabetes or type 2 diabetes, particularly to compare psychological and psychoeducational interventions, and to compare peer support with alternative approaches. Future studies should prespecify diabetes distress as an outcome and provide complete quantitative data to support robust evidence synthesis. Strengthening this evidence base will enable future guidelines to make more definitive recommendations regarding intervention choice and resource allocation.

### AID systems

It is apparent that the development and evaluation of AID systems is moving rapidly. We recommend that all new trials of AID systems include a measure of diabetes distress to determine the effects (direct or indirect) of advanced technologies on the emotional burden of diabetes. This will ensure that future revisions of this guideline are informed by a strong evidence base.

### Diabetes distress as a primary outcome

Many of the RCTs that informed this guideline were designed to assess the impact of the interventions on glycaemic outcomes rather than diabetes distress. We recommend that future RCTs examine diabetes distress as a primary or co-primary outcome or, when this is not feasible, prioritise its reporting as a secondary outcome. This would ensure that RCTs adhere to the principles of the person-centred core outcome set, developed to standardise personalised diabetes care across European health settings [169]. It would also guarantee that trials are specifically designed and adequately powered to detect changes in diabetes distress. Future RCTs should be based on validated minimal clinically important differences in diabetes distress.

The reporting of diabetes distress in RCTs should also be improved. In current trials there is inconsistent use of validated diabetes distress measures and variability in timing, further complicating the evidence synthesis. Trials should include longer follow-up periods to determine whether effects are maintained over time and to ensure that future meta-analyses can compare outcomes at specific time points.

### Contextual factors

The evidence base should be sufficiently sophisticated to inform optimal implementation. This means adapting trials to include contextual factors, such as cultural and ethnic diversity, available health system resources and the preferences of people living with diabetes. In particular, there is a need for RCTs involving South Asian, Middle Eastern and African populations.

Implementation research should examine how interventions can be adapted to diverse cultural and healthcare contexts, ensuring scalability and equity. Finally, although a reduction in diabetes distress is important per se, longitudinal studies are needed to determine whether reductions in diabetes distress translate into sustained improvements in clinical outcomes, such as glycaemic management and reductions in diabetes-related complications. Addressing these priorities will strengthen the evidence base, enabling future guidelines to include recommendations that reflect both efficacy and real-world applicability.

### Cost-effectiveness

Given the limited evidence on the cost-effectiveness of interventions targeting diabetes distress, further research is needed to examine their economic value across diverse healthcare settings.

### Training of healthcare professionals

Good Practice Statements were developed for the assessment of diabetes distress based on realist reviews [56, 57]. For future revisions of this guideline, evidence is needed on the development and effectiveness of training programmes for healthcare professionals, including supervision arrangements and team-based models of clinical care.

## Implementation

Implementing this guideline will require a comprehensive, multi-level approach to ensure relevance, equity and sustainability. Although the guideline has been developed in Europe and many of its principles will apply worldwide, adaptation will be necessary for use in different healthcare settings. For example, many health services do not have sufficient psychologists (with expertise in diabetes) to deliver psychological interventions, while CGM is not universally available. In addition, the concept of person-centred care may need modification in some parts of the world. The EASD supports the process of adolopment—adoption, adaptation and development of the guideline for local contexts [170]. This includes cultural adaptation to reflect differences in health systems, languages and beliefs, and addressing social disparities related to ethnicity, socioeconomic deprivation and health literacy to promote equitable access to psychosocial care.

Successful implementation will depend on raising awareness of the emotional aspects of diabetes and embedding these into routine care. People with diabetes have a central role in ensuring that assessment of diabetes distress becomes standard practice, and they should be supported through the provision of plain-language summaries, practical tools and self-management resources.

A skilled workforce is essential for implementation, and engagement with healthcare professionals should occur at multiple levels. At an individual level, healthcare professionals should be empowered through training, including continuing professional development and integration of psychosocial care principles into undergraduate and postgraduate curricula. Ideally, diabetes care should be characterised by multidisciplinary teams involving specialist diabetes doctors, nurses, psychologists, dietitians and primary care teams. However, even in high income countries, multidisciplinary care is unevenly implemented, despite recommendations from bodies such as the UK National Institute for Health and Care Excellence and the ADA. In low- and middle- income countries, multidisciplinary care is seldom available. Interdisciplinary collaboration should be fostered, and organisational culture and values should support multidisciplinary, person-centred care that integrates psychosocial and biomedical aspects of diabetes management, supported by leadership and capacity building. At a national level, advocacy by professional bodies and diabetes organisations will be required to secure policy support and resources for implementation.

To facilitate uptake, local teams should develop practical implementation tools such as assessment templates, care pathways, examples of best practice and business cases. Digital health solutions and telemedicine can serve as important enablers, improving access to psychosocial support and facilitating guideline integration into routine care. Sustainability will depend on embedding recommendations into clinical workflows, electronic health records and quality improvement cycles, supported by monitoring and feedback mechanisms. By combining cultural adaptation, education, system-level advocacy and empowerment, this guideline will enable recommendations to inform real-world impact.

## Discussion

This guideline represents a significant milestone in the assessment and management of diabetes distress in clinical practice, and is notable as the first evidence-based clinical practice guideline issued by the EASD. Developed through a rigorous, transparent and inclusive process, the guideline provides evidence-based recommendations for the assessment and management of diabetes distress among adults with type 1 diabetes and type 2 diabetes. It addresses a long-standing gap in diabetes care by offering practical guidance to healthcare professionals seeking to integrate emotional support into routine clinical practice.

In terms of the assessment of diabetes distress, eight Good Practice Statements highlight the importance of recognising that emotional health, and the care required to support it, is as important as physical health and the care required to support that. By encouraging open, empathic conversations (A.1, A.2) and the use of validated tools to identify diabetes distress (A.3), these recommendations provide healthcare professionals with opportunities to understand the challenges of living with and managing diabetes every day. Regular monitoring, at least annually (A.4), ensures that emotional needs are not overlooked. Acknowledging and discussing responses to assessments (A.5) builds trust, validates experiences and supports person-centred care. Recording and communicating these findings across the multidisciplinary team (A.6) facilitates continuity of care and collaboration in care planning. Importantly, developing a joint plan with the person experiencing diabetes distress (A.7) also promotes person-centred care. Finally, general and diabetes-specialist healthcare professionals should have the competency to offer psychological support or refer to specialist support when it is needed (A.8). Together, these practices create a structured and compassionate approach to integrating the emotional side of diabetes into high-quality diabetes care.

For the management of diabetes distress, the GRADE recommendations reflect a cautious approach to selecting interventions to support adults with type 1 diabetes experiencing diabetes distress, highlighting both the potential and the limitations of the evidence base. Within technology-based interventions, the recommendations support the use of CGM rather than capillary glucose monitoring (M1.5), while suggesting a small potential benefit of AID systems for diabetes distress (M1.6). Psychological interventions are recommended in addition to usual care, rather than usual care alone, for adults with type 1 diabetes (M1.1). It was difficult to recommend psychoeducational or educational interventions as effective strategies to reduce diabetes distress because of the paucity of RCTs. Similarly, the evidence does not support substituting one type of psychosocial intervention for another (M1.3, M1.4), which may indicate the need for nuanced, person-centred care, tailored to individual needs, priorities and preferences. Taken together, these recommendations show that, while some strategies, especially psychological interventions and CGM, are likely to be effective for reducing diabetes distress in adults with type 1 diabetes, many commonly considered interventions lack a robust evidence base, reinforcing the importance of ongoing research and individualised, holistic clinical care.

Among adults with type 2 diabetes, several psychosocial and educational interventions may help to reduce diabetes distress. Overall, however, the quality of the evidence base remains low to very low, suggesting that the recommendations need to be considered with some caution. Psychological, psychoeducational and educational interventions offer benefits for reducing diabetes distress when added to usual care (M2.1–2.3). Peer support interventions alone are not recommended for the specific purpose of reducing diabetes distress (M2.4), given the absence of a clear effect compared with usual care. Psychological, psychoeducational and educational interventions may be used interchangeably (M2.5–2.7), suggesting that person-centred approaches can be considered without compromising effective intervention. Unlike in type 1 diabetes, there is no consistent evidence that CGM reduces diabetes distress among adults with type 2 diabetes (M2.8), which may reflect differences in management needs and the day-to-day burden between the two conditions. Overall, these GRADE recommendations emphasise the value of integrating psychological and educational strategies into routine care to support adults with type 2 diabetes experiencing diabetes distress. They also highlight the pressing need for stronger, higher quality studies to guide future practice with greater certainty.

### Strengths and limitations

A key strength of this guideline is its robust foundation in both evidence and lived experience. The inclusion on the GDP of people living with diabetes ensured that the recommendations reflect real-world needs and priorities. The use of GRADE methodology and the commissioning of a de novo systematic review of RCTs to provide evidence to inform the management recommendations were critical to ensuring the credibility and applicability of the recommendations. By drawing on findings from realist reviews to formulate Good Practice Statements for the assessment of diabetes distress, the GDP recognised the value of conceptual, observational and experiential evidence, rather than RCTs, for informing some aspects of clinical care.

The guideline highlights the diversity of interventions available for supporting people experiencing diabetes distress, including psychological, psychoeducational and educational approaches, but the evidence base remains limited in several areas. Many recommendations are conditional and based on low or very low certainty of evidence. This reflects multiple factors across the GRADE domains, including imprecision, statistical and clinical heterogeneity, and methodological limitations of individual trials, and highlights the need for further high-quality research. Moreover, diabetes distress was often not specified as a primary outcome, and head-to-head comparisons between distinct intervention types were limited. Data on long-term effects were sparse, and evidence from under-represented populations and healthcare settings remains insufficient.

Furthermore, the guideline focused on comparisons between distinct intervention types or between interventions and usual care and did not evaluate the additive effects of layering one intervention component on top of another (e.g. peer support provided in addition to diabetes self-management education or to other psychological or educational interventions). Psychoeducational interventions were treated as a distinct intervention type a priori and were evaluated as such, rather than as add-on combinations. However, the boundaries between psychological, psychoeducational and educational interventions were sometimes difficult to delineate in practice, as many interventions included overlapping relational or behavioural components. In addition, comparisons between interventions sharing the same overarching intervention type were not undertaken, as such contrasts are not suitable for structured quantitative synthesis and interpretation.

An important limitation of the guideline is related to the scope of the evidence base for interventions. While RCTs are considered the most reliable design for evaluating the effectiveness of interventions, complementary approaches such as qualitative or mixed-methods research may provide additional insights into how support for diabetes distress is experienced and implemented in routine care. It is important to recognise that an absence of RCT evidence should not be interpreted as evidence that an intervention is ineffective. Such interventions may still offer benefits for diabetes distress and other outcomes that were outside the scope of this guideline. This is particularly the case for psychological or psychoeducational interventions. As many of these studies did not focus specifically on diabetes distress, reporting of this outcome was inconsistent or available only in supplementary materials. Consequently, some studies could not be included in quantitative synthesis despite reporting relevant findings.

More rigorous trials with diabetes distress as a prespecified outcome and with transparent and complete outcome reporting are needed to strengthen the evidence base and inform future recommendations.

### Equity, diversity and inclusion

Individual differences in beliefs, abilities, preferences and backgrounds are acknowledged, although limited data meant that not all backgrounds and cultures could be explicitly represented. The Good Practice Statements and recommendations may therefore not be applicable in all cultural or contextual settings, and tools for assessing diabetes distress may not always be practical or feasible. The systematic review informing this guideline included studies from 39 countries across all IDF regions. Of the 248 studies, 30 were conducted exclusively in low- and middle-income countries. These studies encompassed diverse populations in terms of culture, ethnicity, healthcare setting and diabetes stage. However, most studies were conducted in Europe, North America and the Western Pacific, with fewer studies from South America, the Middle East and North Africa, Africa and South-East Asia. Culturally specific examples were omitted to avoid making potentially invidious choices about inclusion.

### Conclusion

In conclusion, this guideline offers a timely and essential framework for addressing diabetes distress. By embedding emotional support into routine care, it has the potential to improve both the experiences and the outcomes of people living with diabetes. Continued research, investment in implementation and commitment to person-centred care will be critical to realising the full impact of the EASD’s first evidence-based clinical guideline, which focuses on the assessment and management of diabetes distress in adults with type 1 diabetes or type 2 diabetes.

## Supplementary Information

Below is the link to the electronic supplementary material.

Supplementary file1 (CRDOWNLOAD 6987 KB)

## Data Availability

All data extracted and analysed for the clinical practice recommendations of this guideline are available in the electronic supplementary material. These data were based on systematic and realist reviews. For access to additional data from the systematic review, which informed the management questions, contact Associate Professor Femke Rutters (email: rutters@amsterdamumc.nl); for access to additional data from the realist reviews, which informed the assessment questions, contact Professor Jackie Sturt (email: jackie.sturt@kcl.ac.uk).

## Abbreviations

AID: Automated insulin delivery
CBT: Cognitive behavioural therapy
CGM: Continuous glucose monitoring
DDS: Diabetes Distress Scale
EtD: Evidence to Decision
GDP: Guideline Development Panel
GOC: Guidelines Oversight Committee
GRADE: Grading of Recommendations, Assessment, Development, and Evaluation
PAID: Problem Areas in Diabetes
PICOT: Participants, Intervention, Comparator, Outcomes, Type of study
PROM: Person-reported outcome measure
SMD: Standardised mean difference
